# MicroRNA-6498-5p Inhibits *Nosema bombycis* Proliferation by Downregulating *BmPLPP2* in *Bombyx mori*

**DOI:** 10.3390/jof7121051

**Published:** 2021-12-08

**Authors:** Congwu Hu, Zhanqi Dong, Boyuan Deng, Qin Wu, Peng Chen, Cheng Lu, Minhui Pan

**Affiliations:** 1 State Key Laboratory of Silkworm Genome Biology, Southwest University, Chongqing 400716, China; 18013127742@126.com (C.H.); zqdong@swu.edu.cn (Z.D.); dengboyuan99@163.com (B.D.); wuuqin@163.com (Q.W.); pjchen@swu.edu.cn (P.C.); 2 Key Laboratory of Sericultural Biology and Genetic Breeding, Ministry of Agriculture and Rural Affairs, Southwest University, Chongqing 400716, China

**Keywords:** *B. mori*, *N. bombycis*, microRNA, miR6498-5p, *BmPLPP2*, anti-microsporidian

## Abstract

As microRNAs (miRNAs) are important expression regulators of coding RNA, it is important to characterize their role in the interaction between hosts and pathogens. To obtain a comprehensive understanding of the miRNA alternation in *Bombyx mori* (*B. mori*) infected with *Nosema bombycis* (*N. bombycis*), RNA sequencing and stem-loop qPCR were conducted to screen and identify the significantly differentially expressed miRNAs (DEmiRNAs). A total of 17 such miRNAs were identified in response to *N. bombycis* infection, among which miR6498-5p efficiently inhibited the proliferation of *N. bombycis* in BmE-SWU1 (BmE) cells by downregulating *pyridoxal phosphate phosphatase 2* (*BmPLPP2*). In addition, a fluorescence in situ hybridization (FISH) assay showed that miR6498-5p was located in the cytoplasm of BmE cells, while it was not found in the schizonts of *N. bombycis*. Further investigation of the effect of *BmPLPP2* on the proliferation of schizonts found that the positive factor *BmPLPP2* could facilitate *N. bombycis* completing its life cycle in cells by overexpression and RNAi of *BmPLPP2*. Our findings offer multiple new insights into the role of miRNAs in the interaction between hosts and microsporidia.

## 1. Introduction

Microsporidia are obligate intracellular parasites that infect multiple species of invertebrates and vertebrates, including humans. These parasites can lead to huge economic losses to sericulture, cause colony mortality in honey bees, and cause health concerns in human patients with immunodeficiency [1,2]. Microsporidia have an extremely reduced genome ranging from 2.3 to 23 Mb, indicating an extreme dependence of the parasite on the host for biochemical processes [3,4]. There have been significant advances in the parasitological, evolutionary, and genomic research on microsporidia since multiple genome sequences were sequenced [5,6,7,8,9,10,11,12,13]. In addition, transcriptomics analysis of microsporidia infection in hosts has revealed a complicated and interconnected interaction in which microRNA (miRNA) as a key gene expression regulator is involved. However, the functions of most miRNAs in the host-microsporidia interaction remain unclear.

MiRNA are a class of small RNA molecules that were first found in *Caenorhabditis. elegans* by functional characterization of *lin-4*. This RNA can be processed into a 22 nt single-stranded RNA, and it has a post-transcriptional regulatory function via incomplete complementary binding with the 3′UTR region of the target gene [14]. To date, more than 30,000 miRNAs have been found in multiple species, including animals, plants, and microbes. The functional characterization of miRNAs in host–pathogen interactions has been reported in the field of host–virus interactions. The host miRNAs can influence viral replication and pathogenesis by direct binding to the RNA virus genome or by causing alterations in the host transcriptome by targeting host genes [15,16]. MiRNA also plays an important role in regulating host internal homeostasis through miRNA–microbiota interactions [17]. Comparatively few studies have reported miRNA-mediated host–fungus interactions. Some plants are able to secrete miRNAs and transport them into the pathogen via an indistinct mechanism, thereby reducing the virulence of the pathogen and enhancing the resistance of the host [18]. As a fungus-like pathogen, a comprehensive investigation of miRNA-mediated regulation of microsporidia could provide insights into the host-microsporidia interaction.

As a model organism of Lepidoptera, silkworm is not only widely used in scientific research, but also one of the important economic animals in the world. *N. bombycis* infects silkworms and causes Pebrine, a chronic disease that results in significant damage to sericulture. As the first discovered microsporidian parasite, the *N. bombycis* genome has been sequenced [19]. As a parasite, *N. bombycis* is an ideal model for research on the host-microsporidia interaction due to the lack of infectivity to human beings and the ease of construction of an *N. bombycis*-infected cell model. The functional characterization of miRNA in the interaction between *B. mori* and *N. bombycis* could provide a reference for research into the mechanism of microsporidian infection and host defence and the regulatory role of key miRNAs in this interaction.

To elucidate the regulatory mechanism of miRNA in response to *N. bombycis* infection in *B. mori*, we identified the DEmiRNAs in *N. bombycis* infected BmE cells and explored the mechanism of DEmiRNAs in the regulation of *N. bombycis* proliferation. In this study, we identified 17 DEmiRNAs in infected BmE cells at 48 h by RNA sequencing and stem-loop qPCR. The *β-tubulin* of *N. bombycis* had a higher expression level at 48 h than at other time points, and immunofluorescence analysis suggested a rapid proliferation of schizonts at 48 h. A highlighted miRNA, miRNA6498-5p, was further studied regarding its functional characterization and the mechanism of miR6498-5p inhibiting the proliferation of schizonts by downregulating *BmPLPP2*. Our results indicated a significant regulatory role of host miRNA in the defense against the pathogen.

## 2. Materials and Methods

### 2.1. Sample Preparation

BmE-SWU1 cells were maintained at 27 °C in Grace Insect medium supplemented with 10% (*v*/*v*) fetal bovine serum (Gibco, Waltham, MA, USA). *N. bombycis* CQ1 strain was conserved in the China Veterinary Culture Collection Center (CVCC No. 102059). Mature spores were germinated in 0.1 M KOH solution and were then added to BmE cells for infection at a ratio of 10–30:1 Cell samples were collected and sent for RNA sequencing at 48 h after infection with *N. bombycis.*

### 2.2. RNA Sequence and Data Analysis

RNA sequence and data analysis were performed as described previously [20]. A certain range of length from clean reads was chosen for downstream analyses. The small RNA tags were mapped to *B. mori* reference sequences by Bowtie without mismatch, and the mapped small RNA tags were used to search for known miRNAs by using miRBase20.0 as a reference [21]. The available software programs miREvo andmirdeep2 were integrated to predict novel miRNAs through exploring the secondary structure, the Dicer cleavage site, and the minimum free energy of the small RNA tags unannotated in the former steps [22,23].

### 2.3. Quantification of miRNA and Target Gene Prediction of miRNAs

Quantification of miRNA expression levels was estimated as TPM (transcripts per million) [24]. Differential expression analysis of samples was performed using the DEGseq (2010) R package. The *p*-values were adjusted using qvalues [25]. A q value < 0.01 and |log2 (fold change)| > 1 were set as the threshold criteria for significantly differential expression by default; afterward, stem-loop qPCR was adopted to validate the expression levels of significantly differentially expressed miRNAs (DEmiRNAs). Prediction of the target genes of miRNAs was performed by miRanda-3.3a [26]. We used KOBAS software to statistically test the enrichment of the target gene candidates in KEGG pathways [27].

### 2.4. Real-Time Quantitative Polymerase Chain Reaction (qPCR)

Cell samples were treated with lysis buffer, and miRNA was separated from total RNA by using a MolPure^®^ Cell/Tissue miRNA Kit (Yeasen, Shanghai, China). For qPCR, specific stem-loop primers were used for reverse transcription following the instructions of a PrimeScript^TM^RT reagent kit with gDNA Eraser (Takara, Beijing, China), and qPCR reactions were performed in a CFX96 Real-Time System using NovoStar SYBR qPCR SuperMix plus (Jinan, China). The qPCR program was as follows: 95 °C for 30 s, 40 cycles of 95 °C for 5 s, and 60 °C for 30 s. Data were analyzed using snRNA U6 and sw22934 (*B. mori* eukaryotic translation initiation factor 4A) as an endogenous control to quantify the expression levels of miRNA and mRNA, respectively, using the 2^−ΔΔCt^ method [28]. Meanwhile, *small subunit* *ribosomal RNA* gene copies of *N. bombycis* were detected by qPCR to examine their proliferation in BmE cells.

### 2.5. Vector Construction

To explore the functions of DEmiRNAs, the U6 promoter of *B. mori* was used to initialize the expression in BmE cells. In addition, a heterogenous RNA with a length of 21 nt was synthesized as a negative control (NC), and miRNA expression cassettes were cloned into pSL1180 plasmids and sequenced for use. Specific miRNA inhibitors were synthesized by the Tsingke Biotechnology Company (Beijing, China). The ORF of *BmPLPP2* was cloned into a pIZV5/HIS vector and named pIZ-*BmPLPP2*. Related primers and RNA sequences are listed in the Supplementary Table S1.

### 2.6. Fluorescence In Situ Hybridization (FISH)

To clearly identify the location of miR6498-5p in BmE cells infected by *N. bombycis* at 48 h, a digoxin-labeled specific probe was synthesized, and a miRNA fluorescence in situ hybridization (FISH) assay was conducted according to the instructions of a D-T-G type miRNA in situ hybrid kit. *N. bombycis* *β-tubulin* rabbit polyclonal antibody was used to locate the positions of schizonts after hybridizing the probe to the slide. All slides were examined under a super resolution laser Scanning confocal microscope with the proper filter (Olympus, Tokyo, Japan).

### 2.7. Western Blotting

Cell samples were lysed in RIPA lysis buffer (Biyuntian, Beijing, China) containing phenylmethanesulfonyl fluoride (PMSF) with a final concentration of 1 mM, and lysates were centrifuged at 12,000× *g* for 10 min at 4 °C. The supernatant was collected, and the concentration of total proteins was quantified by the BCA Protein Assay (Biyuntian, China) after centrifugation. Samples were separated by SDS-PAGE and transferred to a polyvinylidene fluoride (PVDF) membrane (Millipore, Middlesex County, MA, USA). The α-tubulin of *B. mori* was used as an endogenous control to quantify the expression level of α-PTP2 of *N. bombycis.* HRP-conjugated anti-rabbit IgG (1:5000) was used as a secondary antibody. The bands were visualized using a Clarity Western ECL Substrate kit (Biyuntian, China).

### 2.8. Dual Luciferase Assay

The binding sequence of miR6498-5p on *BmPLPP2* mRNA was predicted, then cloned into pGL3-IE1-FLuc and named pGL3-WT-*BmPLPP2*-Luc. The binding site to seed sequences 1–6 of miR6498-5p were mutated to the complementary bases and then cloned into pGL3-IE1-FLuc and named pGL3-MUT-*BmPLPP2*-Luc. Renilla luciferase was used as an endogenous control to calibrate the activities of firefly luciferase expressed by pGL3-IE1-RLuc. BmE cells were co-transfected with FLuc reporter vector, miR6498-5p expression vector, and RLuc reporter vector at a ratio of 4:4:1. The luciferase activities were detected by a Dual-Glo Luciferase Assay System (Promega, Madison, WI, USA) at 48 h post transfection, and the values of FLuc/RLuc were calculated.

### 2.9. Statistical Analysis

All data are shown as the mean ± standard deviation from three independent experiments. All statistical analyses of the values were conducted using a two-tailed unpaired Student *t*-test. Values with * *p* < 0.05 mean significant difference, ** *p* < 0.01 represents very significant difference. All the statistical analyses were performed using GraphPad Prism 7.0 software.

## 3. Results

### 3.1. Characterization of the Proliferation of N. bombycis in BmE Cells

**Figure 1:**
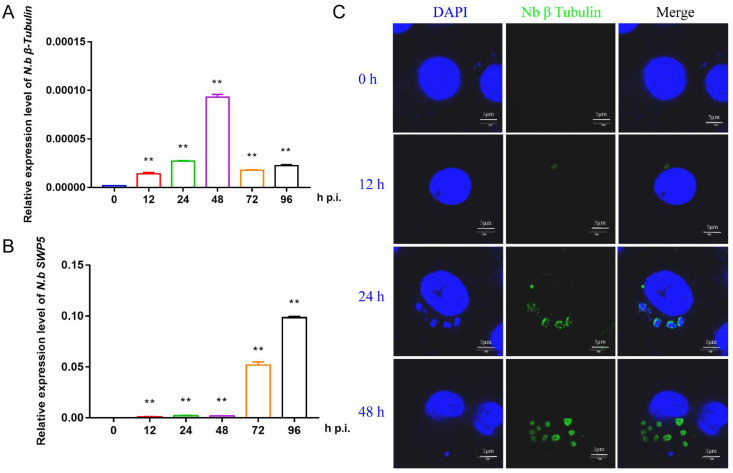
Characterization of the proliferation of *N. bombycis* in BmE cells (**A**) The expression level of the *N. bombycis* *β-tubulin* gene at different time points in BmE cells. (**B**) The expression level of *N. bombycis swp5* at different time points in BmE cells. (**C**) Immunofluorescence analysis of schizont location in BmE cells at different time points. DAPI staining represents cell nuclei, and green fluorescence represents the β-tubulin protein of *N. bombycis*. All data represent the means of three replicates ± SD, **, *p* < 0.01.

To prepare the infected BmE cells for RNA sequencing, we first investigated the proliferation of *N. bombycis* in BmE cells by qPCR and immunofluorescence. Two specific genes, *β-tubulin* and *SWP5*, were chosen for qPCR detection. The results showed that the structural protein gene *β-tubulin* had a relatively high expression level at 48 h, while the spore wall protein gene *SWP5* was significantly upregulated after 48 h, indicating a proliferation period of *N. bombycis* in BmE cells at this time (Figure 1A,B). Immunofluorescence analysis indicated clear amplification of schizonts at 48 h (Figure 1C). Based on the above results, the BmE cells infected by *N. bombycis* at 48 h and normal control cells were sent for RNA sequencing.

### 3.2. Data Analysis

**Figure 2:**
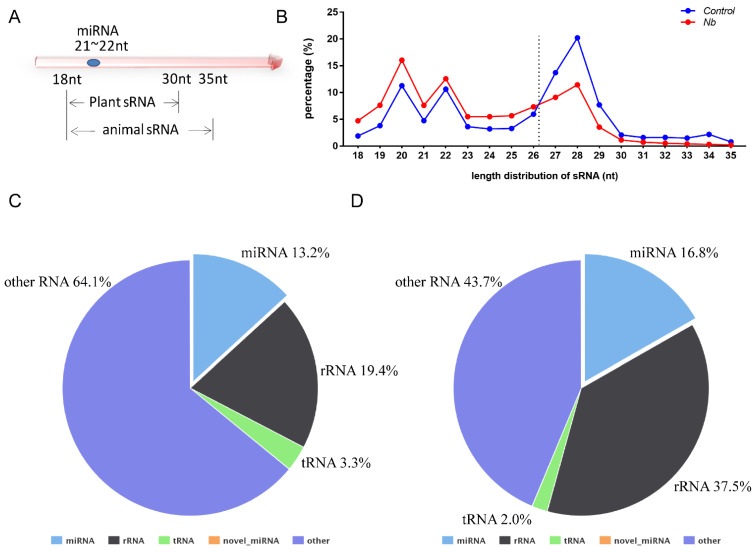
RNA sequence data analysis of BmE cells infected by *N. bombycis* and uninfected cells (**A**) Length range of sRNA in animals and plants. (**B**) Percentage of the sRNA mapped to the reference sequence of two groups. (**C**,**D**) The proportions of known miRNAs, rRNAs, tRNAs and other RNAs in mapped sRNA of the control group and the infected group.

Data analysis was performed as described previously and the percentages of clean reads among raw reads of both groups were above 95% [20]. In general, the sRNA length in animals ranges from 18 to 35 nt, so we chose the sRNA of this section to perform a statistical analysis of the length distribution (Figure 2A). The results showed that there was a clear difference in the distribution of each length of sRNA; the percentage of the sRNA length range from 18 to 26 nt was increased in BmE cells infected by *N. bombycis*. In contrast, the proportion of sRNA with length range from 27 to 35 nt was decreased relative to the uninfected group (Figure 2B). To obtain a comprehensive understanding of the alteration of sRNA, the sRNAs identified as having a length range from 18 to 35 nt were mapped to *B. mori* reference sequences by Bowtie without mismatch. Totals of 16,960,665 (Control group) and 9,023,874 (*N.b* group) sRNA reads with lengths from 18 to 35 nt were obtained, of which 84.16% and 77.85%, respectively, were mapped to reference sequences. To obtain a unique annotation for each sRNA, the sRNAs that were mapped to the reference sequences were annotated in the order of known miRNA > rRNA > tRNA > snRNA > snoRNA > repeat > gene > novel miRNA. The results showed that the proportions of rRNA in both groups were lower than 37.5%, indicating that our data were qualified for further analysis. The proportion of known miRNAs also had an increased tendency in cells that were infected by *N. bombycis* (Figure 2C,D). Our results showed that the sequence data were adequate for further analysis, and the host miRNAs reflected a response to infection by *N. bombycis*.

### 3.3. Expression Analysis and Functional Detection of Host miRNAs

**Figure 3:**
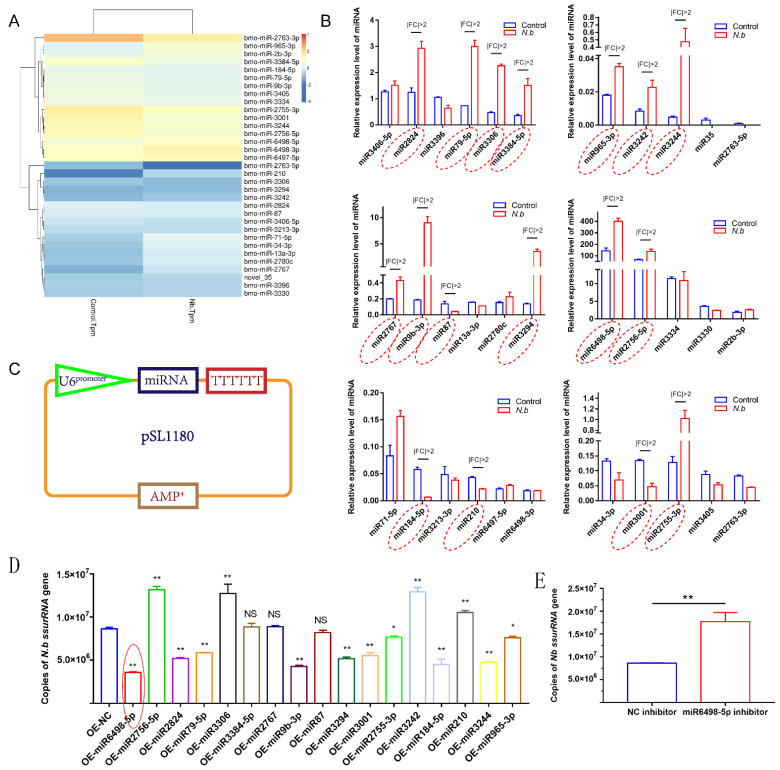
Validation of DEmiRNAs and the effect on *N. bombycis* proliferation (**A**) Heat map of DEmiRNAs screened by RNA sequence. (**B**) Stem-loop qPCR analysis of DEmiRNAs alteration after the infection with *N. bombycis*. (**C**) Vector construction for the expression of DEmiRNAs. (**D**) Copy analysis of *N. bombycis* ssurRNA gene in BmE cells transfected with different miRNA expression vectors by real-time quantitative polymerase chain reaction (qPCR) at 48 h post-infection. (**E**) Analysis of *N. bombycis* proliferation in BmE cells transfected with the inhibitor of miR6498-5p. All data represent the means of three replicates ± SD, **, *p* < 0.01, NS, *p* > 0.05.

To identify the miRNA response to infection in BmE cells, the expression levels of miRNAs were estimated as TPM (transcripts per million). A total of 33 miRNAs were differentially expressed with a q value < 0.01 and |log2 (fold change)| > 1, among which 11 miRNAs were upregulated and 22 were downregulated (Figure 3A). Following the identification, stem-loop qPCR was conducted to validate the expression levels of the 33 miRNAs. The results showed that 17 miRNAs were identified to have different expression with |fold change| > 2 (Figure 3B).

To explore the functions of these 17 DEmiRNAs in *N. bombycis* proliferation, we constructed expression vectors for the 17 DEmiRNAs. The U6 promoter and TTTTTT were used for miRNA transcription and termination, respectively (Figure 3C). All vectors were sequenced prior to use. Then, BmE cells were infected with *N. bombycis* after being transfected with the DEmiRNA expression vector. The copy numbers of the *N. bombycis small subunit rRNA* (*ssurRNA*) gene in BmE cells were detected by qPCR at 48 h post infection, and the results showed that 14 DEmiRNAs had various effects on the proliferation of schizonts, among which four miRNAs had a stimulative effect on *N. bombycis,* while the others could inhibit the proliferation of schizonts in BmE cells. In particular, miR6498-5p displayed more effective inhibition of the replication of the *ssurRNA* gene (Figure 3D). In contrast to the inhibitory effect of miR6498-5p, the inhibitor of miR6498-5p significantly promoted the proliferation of schizonts (Figure 3E). Our results provide a comprehensive understanding of the response and regulation of host miRNA to *N. bombycis* infection.

### 3.4. Functional Characterization and Location of miR6498-5p in BmE Cells

**Figure 4:**
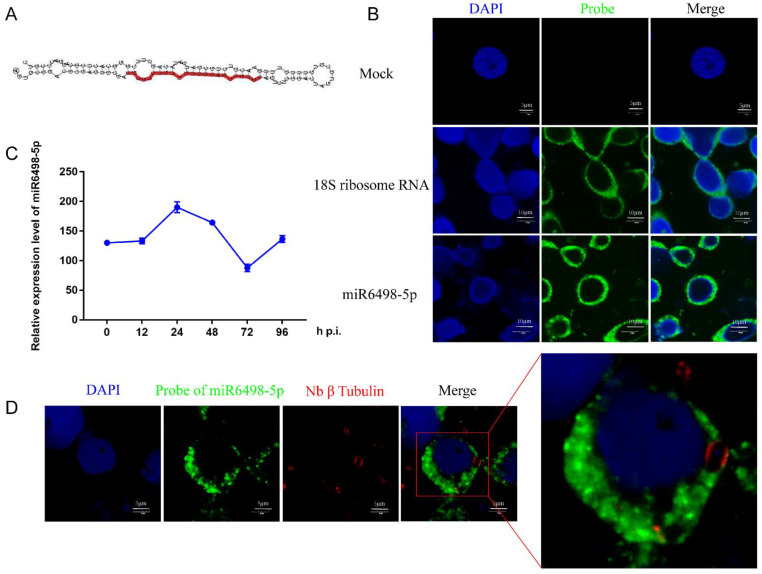
Characterization of miR6498-5p in BmE cells (**A**) Precursor structure of miR6498-5p. The red color represents the location and sequence of miR6498-5p. (**B**) Fluorescence in situ hybridization (FISH) of miR6498-5p and *B. mori* 18S RNA. The DAPI staining represents the nuclei and green fluorescence represents the location of miR6498-5p or 18S ribosomal RNA. (**C**) Alteration of the expression level of miR6498-5p with the infection of *N. bombycis* at different time points. (**D**) The location of miR6498-5p in BmE cells infected by *N. bombycis*. The green fluorescence represents the location of miR6498-5p, and the red fluorescence represents the locations of schizonts.

MiR6498-5p is a sRNA with a length of 23 nt, and the precursor has a typical hairpin structure. The mature sequence of miR6498-5p is shown in Figure 4A. The qPCR analysis found that the expression pattern of miR6498-5p was similar to that of *N. bombycis* *β-tubulin* in infected cells, suggesting a close connection between the *B. mori* and *N. bombycis* (Figure 4C). It has been reported that host miRNAs could be transported to pathogens and target the genes of the pathogens. To explore whether miR6498-5p could target the genes of *N. bombycis*, and then inhibit its proliferation, a FISH assay was conducted to confirm the location of miR6498-5p in infected cells. The results showed that miR6498-5p was located in the cytoplasm of BmE cells, with the same locations as 18s rRNA (Figure 4B). In the infected cells, miR6498-5p was present in the BmE cells but could not be found in the schizonts (Figure 4D), indicating that miR6498-5p inhibited the proliferation of *N. bombycis* by targeting the genes of BmE cells.

### 3.5. Regulation of the Target Gene by miR6498-5p

**Figure 5:**
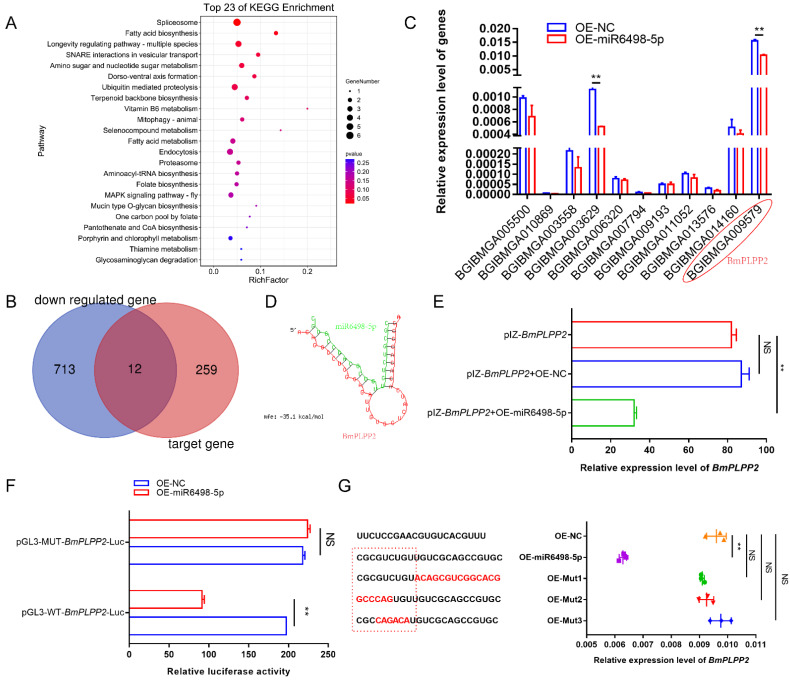
Identification of how miR6498-5p negatively regulates *BmPLPP2* (**A**) KEGG enrichment analysis of the target genes of miR6498-5p predicted by miRanda-3.3a. (**B**) Venn diagram depicting the genes targeted by miR6498-5p and significantly downregulated by *N. bombycis* infection. (**C**) Expression of 12 genes obtained from the Venn diagram in BmE cells transfected with an OE-miR6498-5p vector. (**D**) The predicted binding structure of miR6498-5p with *BmPLPP2* by RNA22. Green represents the miRNA, and red represents the mRNA. (**E**) qPCR analyzes the expression of *BmPLPP2* in BmE cells by co-transfection with the expression vectors of *BmPLPP2* and miR6498-5p (**F**) Dual luciferase assay analysis of the binding of miR6498-5p to the *BmPLPP2*. (**G**) qPCR analyzes the expression of *BmPLPP2* in BmE cells by transfection with the mutants of miR6498-5p. Red sequences represent mutated bases. The dotted red line box represents the seed sequence of miR6498-5p. All data represent the means of three replicates ± SD, **, *p* < 0.01, NS, *p* > 0.05.

To identify the target of miR6489-5p, potential genes were predicted by miRanda-3.3a. A total of 271 genes were obtained with the criterion of minimum free energy < −20 kcal/mol. To obtain comprehensive knowledge concerning the functions of these target genes, KEGG enrichment analysis was performed by KOBAS (http://kobas.cbi.pku.edu.cn/ (25 December 2020)). The bubble diagram (Figure 5A) shows the top 23 KEGG enrichment pathways, among which the top nine pathways were involved in spliceosome, fatty acid biosynthesis, longevity regulating pathway, SNARE interactions in vesicular transport, amino sugar and nucleotide sugar metabolism, dorso-ventral axis formation, ubiquitin mediated proteolysis, terpenoid backbone biosynthesis, and vitamin B6 metabolism.

To find the genes that were regulated by miR6498-5p, we aimed to identify those genes that had a predicted binding site with miR6498-5p and could simultaneously be downregulated by *N. bombycis* infection. According to the transcriptome data, a total of 725 genes were significantly downregulated, among which 12 genes had a potential binding site with miR6498-5p (Figure 5B). To examine the regulation of miR6498-5p on the expression of the 12 genes, the expression levels were detected in BmE cells transfected with the expression vector for miR6489-5p. The results showed that two genes were downregulated with a fold change >2 relative to the control group (Figure 5C).

To validate that *BmPLPP2* (BGIBMGA009579) was the target gene of miR6498-5p, RNA hybrid prediction and dual luciferase assays were performed. The results showed that the binding site of miR6498-5p was located in the ORF of *BmPLPP2,* and the seed sequence of the miRNA (1–9 nt) was completely complementary with the target sequence. The predicted binding structure of miR6498-5p with *BmPLPP2* is shown in Figure 5D. The expression of miR6498-5p significantly decreased the expression of *BmPLPP2* as shown by co-transfection of the expression vectors of miR6498-5p and *BmPLPP2* in cells (Figure 5E). The dual luciferase assay showed that miR6489-5p decreased the value of FLuc/RLuc in the wild-type group, indicating negative regulation of miR6498-5p on *BmPLPP2* (Figure 5F). Furthermore, a series of miR6498-5p mutants was constructed, and the regulatory effect of the mutants on *BmPLPP2* expression disappeared (Figure 5G). Our results validated *BmPLPP2* as being the target gene of miR6498-5p.

### 3.6. BmPLPP2 Promotes Schizont Proliferation

**Figure 6:**
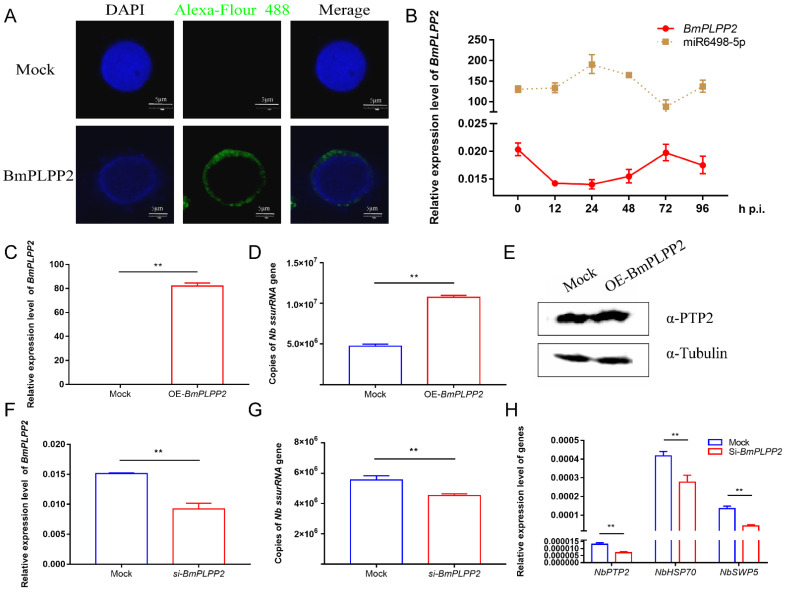
*BmPLPP2* promotes the proliferation of *N. bombycis* (**A**) Expression alteration of *BmPLPP2* after the infection of *N. bombycis* at different time points. (**B**) Immunofluorescence analysis of the location of *BmPLPP2*. DAPI staining represents the nuclei, and green fluorescence represents the location of BmPLPP2. (**C**) qPCR analyzes the expression of BmPLPP2 in BmE cells by transfected with pIZ-*BmPLPP2*. (**D**) Copy analysis of the *N. bombycis* *ssurRNA* gene in BmE cells transfected with pIZ-*BmPLPP2*. (**E**) Western blotting analysis of the expression of *N. bombycis* α-PTP2 in *BmPLPP2* overexpressing cells. (**F**) Expression analysis of *BmPLPP2* in BmE cells transfected with siRNA. (**G**) Copy analysis of the *N. bombycis* ssurRNA gene in BmE cells by transfection with siRNA of *BmPLPP2*. (**H**) Gene expression analyses of *N. bombycisPTP2*, *HSP70* and *SWP5* in BmE cells by transfection with siRNA of *BmPLPP2*. All data represent the means of three replicates ± SD, **, *p* < 0.01.

To investigate the effect of *BmPLPP2* on *N. bombycis* proliferation, the pattern and location of *BmPLPP2* expression were detected. The results showed that *BmPLPP2* was located in the cytoplasm of BmE cells, and it was negatively expressed in relation to miR6489-5p (Figure 6A,B), strongly suggesting that *BmPLPP2* was regulated by miR6498-5p. Moreover, the *ssurRNA* gene copies and α-PTP2 protein of *N. bombycis* were detected by qPCR and Western blotting in *BmPLPP2* overexpressing cells. The results showed that *BmPLPP2* was significantly upregulated, and it significantly promoted the proliferation of schizonts (Figure 6C–E).

To further confirm the effect of *BmPLPP2* on *N. bombycis*, an effective siRNA of *BmPLPP2* was synthesized, and the interference efficiency was detected by qPCR (Figure 6F). The results showed that the *ssurRNA* gene copies were significantly decreased, and the expression levels of *NbPTP2*, *NbHSP70* and *NbSWP5* were inhibited in BmE cells by transfection with the siRNA of *BmPLPP2* (Figure 6G,H). Taken together, the results suggest that miR6498-5p could inhibit *N. bombycis* proliferation by negatively regulating the expression of *BmPLPP2*.

## 4. Discussion

As a fungus-like parasite, microsporidia have become an increasingly important and fascinating model for the study of host-parasite molecular interactions since the discovery of *N. bombycis* in silkworms. At present, the molecular mechanism of the host–microsporidian interaction has been fully characterized, including the regulation of host metabolism, immune evasion, and host defense [29,30,31,32,33]. However, the regulatory mechanisms of miRNA in this relationship are poorly understood. The studies of the regulation of host miRNA on pathogens have great significance for the understanding of host–microsporidian interactions and for parasite research. In our study, 17 miRNAs were identified as having a response to the infection of *N. bombycis*, among which 14 miRNAs had a significant influence on the proliferation of schizonts. In addition, the mechanism of the inhibitory effect of miR6498-5p on *N. bombycis* was elucidated.

Since the discovery of *lin-4*, more than 30,000 miRNAs have been found in multiple species, including animals, plants, and microbes. MiRNA has a post-transcriptional regulatory function via incomplete complementary binding with the 3′UTR region of the target gene. Multiple important processes are regulated by miRNAs, including tumorigenesis, biological development, organ formation, and pathogen defense. Therefore, the functional characterization of miRNAs in their regulatory networks has received widespread attention. In host–virus interactions, the regulatory mechanisms of more than 30 miRNAs in response to various viral infections have been identified. However, few miRNAs have been reported to mediate the interaction between the host and a fungus. Therefore, two specific genes, *N. bombycis* *β-tubulin* and *spore wall protein* 5 were detected in infected cells to identify RNA sequences present in a specific time period (Figure 1). The data showed that the proportion of host miRNA increased from 13.2% to 16.8% after the infection, and further validation found that 17 host miRNAs responded to infection by *N. bombycis* (Figure 2 and Figure 3). Our results provide a comprehensive miRNA alteration reference for research concerning the host response to microsporidian infection.

In the interaction between host and pathogen, the host miRNAs can directly regulate the coding-RNA of pathogens. For example, cotton plants can secrete miR166, andmiR159 and transport these RNAs to *Verticillium dahliae* (a fungal pathogen), thereby enhancing the resistance of the host by downregulating the virulence related genes of the pathogen. Likewise, the pathogen miRNAs also could regulate the host messenger RNAs and thereby act as virulence factors during parasitism, for example in the parasitic plant *Cuscuta* *campestris*. In our research, *N. bombycis* acted as a fungus-like pathogen, and its coding RNA could not be regulated by silkworm miRNA according to the results of the FISH assay of miR6498-5p. Further study found that miR6498-5p could suppress the proliferation of *N. bombycis* by downregulating *BmPLPP2,* indicating indirect miRNA-mediated regulation by the host on microsporidia (Figure 4).

**Figure 7:**
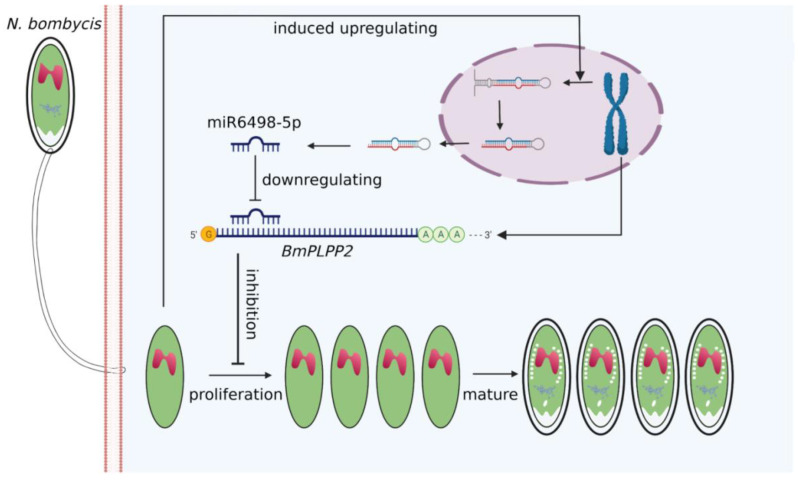
Model for host miR6498-5p inhibits *N. bombycis* proliferation by downregulating *BmPLPP2*.

Due to the special regulation mechanism of miRNA on target genes, there are limits to identifying all the genes that are directly regulated by miRNA. This makes it a significant challenge to obtain a comprehensive functional characterization of miRNAs. In our research, a total of 271 target genes of miR6498-5p were predicted in *B. mori.* The KEGG enrichment analysis of these genes indicated involvement in multiple pathways, including spliceosome, fatty acid biosynthesis, and ubiquitin-mediated proteolysis. To identify the genes that were regulated by miR6498-5p in the interaction between *B. mori* and *N. bombycis*, we screened the genes that had a predicted binding site with miR6498-5p and could simultaneously be downregulated by *N. bombycis* infection. In this way, 12 genes were obtained, and the transcriptional regulation of miR6498-5p on these genes was detected. As a phosphatase, *PLPP* has an in vitro specificity to dephosphorylate pyridoxal 5′-phosphate (PLP) that acts as co-factor in more than 140 different enzyme reactions [34,35,36] and is especially involved in amino acid transport and metabolism. Multiple experiments demonstrated that miR6498-5p could target and negatively regulate *BmPLPP2* and thereby suppress the proliferation of *N. bombycis* (Figure 5 and Figure 6). The results indicated that the host may downregulate the mRNA level of *BmPLPP2* by increasing the expression of miR6498-5p, thus altering the balance of amino acid metabolism to suppress the proliferation of *N. bombycis* (Figure 7). However, how the infection induces the upregulation of miR6498-5p is still unknown. In the upstream region of 20-hydroxyecdysone-responsive miR275 cluster, multiple ecdysone receptor elements have been found, which indicates an inducer-related induction mechanism of miRNA expression [37]. As an inhibitor, the induced upregulation of miR6498-5p may be due to the activation of host pathways involved in response to pathogen invasion. Our research provides an insight into the host response to pathogen infection and miRNA regulation during the host–microsporidian interaction.

## 5. Conclusions

This research identified a total of 17 silkworm miRNAs that response to *N. bombycis* infection, among which 14 miRNAs have significant effects on the proliferation of schizont. Meanwhile, the research also has demonstrated that miR6498-5p suppresses the proliferation of *N. bombycis* by downregulating *BmPLPP2*, an important factor in the regulation of host metabolism. The results have validated the important role of miRNA in the interaction between hosts and fungus-like parasites.

## Data Availability

The RNA-seq data are available in NCBI with accession number: PRJNA760284.

## Supplementary Materials

The following are available online at https://www.mdpi.com/article/10.3390/jof7121051/s1, Table S1: Primers and related RNA sequence used in the study.
